# Management of Subclinical and Overt Hypothyroidism Following Hemithyroidectomy in Children and Adolescents: A Pilot Study

**DOI:** 10.3389/fped.2019.00396

**Published:** 2019-09-27

**Authors:** Jiarui Chen, Shule Hou, Xiaoyan Li, Jun Yang

**Affiliations:** ^1^Department of Otorhinolaryngology-Head and Neck Surgery, Shanghai Children's Hospital, Shanghai Jiaotong University, Shanghai, China; ^2^Department of Otorhinolaryngology-Head and Neck Surgery, Xinhua Hospital, Shanghai Jiaotong University School of Medicine, Shanghai, China; ^3^Ear Institute, Shanghai Jiaotong University School of Medicine, Shanghai, China; ^4^Shanghai Key Laboratory of Translational Medicine on Ear and Nose Diseases, Shanghai, China

**Keywords:** children, hemithyroidectomy, overt hypothyroidism, subclinical hypothyroidism, thyroid hormone replacement

## Abstract

**Background:** To reduce surgical complications and avoid lifelong thyroid hormone replacement, hemithyroidectomy is preferred in children and adolescents with benign nodular thyroid disease. However, hypothyroidism following hemithyroidectomy may occur, and postoperative thyroid hormone replacement for hypothyroidism following hemithyroidectomy is usually administered without a full understanding of the clinical characteristics of hypothyroidism.

**Methods:** To investigate the incidence and risk factors of hypothyroidism after hemithyroidectomy in children and adolescents, and to identify whether postoperative thyroid hormone replacement is necessary, a retrospective review of 43 patients under 18 years of age who underwent hemithyroidectomy from January 2009 to October 2016 was conducted. All hypothyroid patients were retrospectively analyzed to determine the incidence and predisposing factor(s) of postoperative hypothyroidism. All patients were measured regarding age, sex, serum thyrotropin (TSH), anti-thyroid antibody, and histological evidence of lymphocytic infiltration. Hypothyroid patients were measured for symptoms, timing of diagnosis, and thyroid hormone replacement.

**Results:** The mean age at the time of surgery was 13.65 ± 3.04 years. Of the cohort, 34 patients were female (79.07%), and the mean follow-up time was 28 ± 9 months. Hypothyroidism was diagnosed in 11 of the 43 patients. The mean postoperative TSH level was 7.17 ± 2.13 μIU/ml. The mean preoperative TSH level was 3.11 ± 0.59 μIU/ml in hypothyroid patients compared with 1.92 ± 0.72 μIU/ml in euthyroid patients (*P* < 0.05). A preoperative TSH level >2.2 μIU/l and lymphocytic infiltration graded 3 or 4 were found to be independent risk factors for the development of hypothyroidism. There were no significant differences between groups in terms of patient age or sex.

**Conclusions:** In the pediatric and adolescent population, patients with elevated preoperative TSH levels or the presence of lymphocytic infiltration may increase the risk of risk of hypothyroidism. In our study, postoperative levothyroxine (L-T4) treatment was necessary in 16.28% of cases after hemithyroidectomy. Patients with mild postoperative hypothyroidism should be followed up, without the need for immediate L-T4 replacement, so as to expect patients to recover spontaneously.

## Introduction

Hemithyroidectomy is recommended for children and adolescents with unilateral thyroid masses which may cause compression symptoms or cosmetic problems, or to exclude thyroid cancer in the presence of fine-needle aspiration cytology uncertainties. Hypothyroidism is a recognized complication after hemithyroidectomy, and the incidence of benign thyroid disease in adults ranges from 11 to 43% (1–4). Untreated clinical hypothyroidism is associated with many well-known clinical complications, including an increased risk of cardiovascular disease and neuropsychiatric symptoms (5, 6). In infancy and childhood, thyroid dysfunction can adversely affect growth and brain maturation. Moreover, if levothyroxine (L-T4) treatment is inadequate, children with hypothyroidism have an increased risk of impairment with regard to mental retardation, metabolic abnormalities, growth, and skeletal maturation (7).

However, most reports on this subject to date that evaluated pediatric populations have focused on the immediate complications of surgery (laryngeal nerve injury, postoperative bleeding, wound infection) and the malignant tumor rate (8). In contrast, there are no data available on the long-term evolution of thyroid function and risk factors for hypothyroidism following hemithyroidectomy in children and adolescents. Accordingly, this situation motivated us to investigate the incidence of hypothyroidism following hemithyroidectomy in children and adolescents and to identify potential risk factors and the optimal management strategy for patients.

## Patients and Methods

This was a retrospective study of all patients under the age of 18 who underwent hemithyroidectomy, including total thyroidectomy and isthmectomy, for thyroid benign nodular from January 2009 to October 2016. Permission for data acquisition was granted by a protocol approved by Shanghai JiaoTong University Research Compliance Office. All thyroid surgeries were performed by two surgeons at one institution. All patients were preoperatively euthyroid, and the serum thyrotropin (TSH) level was measured at least 6 weeks postoperatively. Patients were excluded from this study for the following: (1) lack of postoperative follow-up or follow-up occurred at another institution; (2) malignant nodular disease or initial total thyroidectomy was performed; (3) actively taking medications known to cause hypothyroidism; (4) previously received radiation therapy to the cervical region; or (5) taking L-T4 prior to surgery.

For all eligible patients, assessments regarding sex and age, the final pathological diagnosis, and the preoperative thyroid function analyses, including the serum TSH level, anti-thyroglobulin antibody (anti-Tg), anti-thyroid peroxidase antibody (anti-TPO), and postoperative serum TSH level, were performed. In our institutional laboratories, the reference ranges of TSH, free thyroxine (FT4) and triiodothyronine (T3) concentrations were defined as 0.6–3.9 μIU/ml, 8.4–15.0, and 3.72–6.61 pmol/l, respectively. Anti-Tg and anti-TPO antibody positivity was defined as levels >4 and >9 U/ml, respectively. Euthyroidism was defined as the presence of normal serum TSH, FT4, and T3 levels. Subclinical hypothyroidism was defined as asymptomatic patients with serum TSH levels above the upper limit of the reference range but with normal FT4 levels. Overt hypothyroidism was defined as symptomatic patients with elevated serum TSH levels and FT4 levels below the reference range. Mean serum TOSH levels were determined in patients with postoperative hypothyroidism before and after surgery and were compared with those of patients with normal thyroid function postoperatively. In all patients, the thyroid lobe extracted during surgery was immediately sent to the laboratory, a single pathologist performed a histological examination of the resected tissue to diagnose the underlying pathology. In addition, the lymphocytic infiltration was ranged from grade 0 to grade 4, as described by Berglund et al. (9). A score of 0 indicated no glandular lymphocytic infiltration, with 1 indicating minimal lymphocytes, and 2 indicating moderate lymphocytic infiltration, and 3 marked diffuse lymphocytic infiltration; a score of 4 was consistent with the histological diagnosis of Hashimoto's thyroiditis, which was characterized by diffuse infiltration and the formation of reproductive centers. During the follow-up period, the development of hypothyroidism, type of hypothyroidism, TOSH level at diagnosis, interval between surgery and development of hypothyroidism, spontaneous recovery from hypothyroidism, interval between the development of hypothyroidism and recovery, and the use of thyroid hormone substitution were recorded.

The statistical software package SPSS, version 18, was used to perform all statistical analyses. The statistical significance of the difference between patients with postoperative hypothyroidism vs. patients with normal thyroid function was determined using Student's *t*-tests and chi-square tests. A *P* < 0.05 was considered significant. Binary logistic regression was performed to evaluate the influence of each variable and variables with *P* < 0.05 for a risk of developing hypothyroidism in univariate analysis. Multivariate analysis was then conducted. The results of the binary logistic regression are presented as the 95% confidence interval (CIs) and odds ratio (ORs) of the *P*-value. Statistical significance was defined as *P* < 0.05, and all *P*-values were bilateral.

## Results

Fifty-one patients undergoing hemithyroidectomy fulfilled the inclusion criteria. Eight patients were not followed up, and 43 were recontacted by telephone to ensure that we had their most up-to-date medical results. Our analyses included 43 patients, 34 females and 9 males, with a mean age of 13.65 ± 3.04 years (range 8–17 years). The mean follow-up period in our study ranged from 19 to 40 months (median of 29 months).

Postsurgical hypothyroidism was found in 11 patients (25.58%). The mean postoperative TOSH level was 7.17 ± 2.13 μIU/ml. Three patients (6.98%) developed clinically overt hypothyroidism. The most common complaints with postoperative clinical hypothyroidism were weakness, fatigue, and weight gain. Eight patients (18.6%) exhibited biochemical subclinical hypothyroidism with an elevated TOSH level, and none of these patients reported any symptoms. Hypothyroidism was diagnosed in nine patients (81.8%) within 3 months after surgery and in two patients (18.2%) within 6 months after surgery. The mean time interval between surgery and the development of hypothyroidism was 4.1 months (range 2–12 months). All three overt hypothyroidism patients were treated with L-T4 immediately after surgery. In addition, four patients showed transient subclinical hypothyroidism with a mildly increased TOSH level was found for the first time within 3 months after surgery, as well as a normal TOSH level recorded within 12 months postoperatively; they were not treated with L-T4. The other four patients with subclinical hypothyroidism received thyroid hormone replacement after 12 months of follow-up. Among the patients who were followed-up, seven (16.28%) were ultimately treated with postoperative L-T4 in order to maintain the normal serum TOSH range. No patient received suppressive doses of L-T4. Furthermore, no patients in our study developed overt or subclinical hypothyroidism after 12 months.

The relationship between clinicopathological characteristics and the development of hypothyroidism after hemithyroidectomy is shown in Table 1. Serum TOSH levels were significantly increased in patients diagnosed with postoperative hypothyroidism. The mean preoperative serum TOSH level in patients with hypothyroidism was also significantly increased. A preoperative TOSH level of at least 2.2 μIU/ml, anti-TPO positivity, and lymphocytic infiltration of grade 3 or 4 were detected to be significantly associated with the development of hypothyroidism in univariate analysis. No significant differences were found in age or sex between the euthyroid and hypothyroid groups. In adjusted multivariate analysis, a preoperative TOSH level of at least 2.2 μIU/ml (OR, 11.812; 95% CI, 1.509–92.44; *P* < 0.05) and lymphocytic infiltration of grade 3 or 4 (OR, 35.593; 95% CI, 1.383–215.975; *P* < 0.05) were confirmed to be independent risk factors for hypothyroidism (Table 2). Although the rate of anti-TPO positivity was significant in univariate analysis, the influence of these factors on the development of hypothyroidism was not found to be significant after adjustment for other factors in multivariate analysis.

**Table 1:** Association between clinicopathological characteristics and development of hypothyroidism after hemithyroidectomy.

	**Hypothyroid (*n* = 11)**	**Euthyroid (*n* = 32)**	***P*-value**
**Sex**			
Female	8	26	0.549 (NS)
Male	3	6	
**Age (y)**	14.6	14.3	
mean ± SD	13.5 ± 3.72	13.7 ± 2.84	0.467 (NS)
≤13.6	5 (45.45%)	14 (43.75%)	0.319 (NS)
>13.6	6 (54.55%)	18 (56.25%)	
**Postoperative TOSH (μIU/ml)**			
mean ± SD	7.17 ± 2.13	2.17 ± 0.86	<0.05
**Preoperative TOSH (μIU/ml)**			
mean ± SD	3.11 ± 0.59	1.92 ± 0.72	<0.05
≤2.2	3 (27.27%)	24 (75%)	<0.05
>2.2	8 (72.72%)	8 (25%)	
**Anti-thyroid antibody positivity**			
Anti-thyroglobulin antibody positivity	9 (81.82%)	26 (81.25%)	0.822 (NS)
Anti-peroxidase antibody positivity	5 (45.45%)	5 (15.6%)	<0.05
**Lymphocytic infiltration**			
0–2	5 (45.45%)	26 (81.25%)	<0.05
3–4	6 (54.55%)	6 (18.75%)	<0.05

*NS, not significant*.

**Table 2:** Univariate and multivariate analyses for the development of hypothyroidism.

	**Univariate**			**Multivariate**		
	**OR**	**95% CI**	***p*****-value**	**OR**	**95% CI**	***p*****-value**
Sex female	1.339	0.279–6.434	0.279	1.457	0.251–8.465	0.675
Age	0.972	0.777–1.216	0.802	0.911	0.713–1.164	0.454
Preoperative TOSH > 2.2 μIU/ml	11.218	2.454–51.284	<0.05	11.812	1.509–92.44	<0.05
Anti-peroxidase antibody positivity	1.722	1.006–2.652	<0.05	1.215	0.952–2.123	0.621
Lymphocytic infiltration (3–4)	37.2	3.662–177.843	<0.05	35.593	1.383–215.975	<0.05

## Discussion

Previous studies have demonstrated an incidence of hypothyroidism ranging from 11 to 43% (1–4). For example, Verloop et al. (2) reported in a meta-analysis that one in five patients developed hypothyroidism after hemithyroidectomy. Our results in children are largely in agreement with the results of other studies. In general, serum TOSH levels should be measured in all patients following hemithyroidectomy to evaluate whether the residual thyroid lobe produces enough thyroid hormones to maintain serum TOSH levels within the normal range. We propose that the postoperative TOSH level should be examined 6 weeks postoperatively and reexamined every 3 months. Because T4 has a half-life of about seven days, early access to postoperative thyroid-stimulating hormone levels is limited; indeed, remaining thyroid function can be accurately assessed only after five half-lives (10). In our study, when hypothyroidism occurred following hemithyroidectomy, it manifested within a mean time of 3.2 months, and the trend in the incidence of hypothyroidism over time suggests that in most cases, it develops during the early postoperative period. In fact, 81.8% of our patients (9/11) were diagnosed within 3 months, and the other 18.2% of patients (2/11) were diagnosed within 6 months. It has been pointed out that hypothyroidism following hemithyroidectomy rarely progresses (1) and that hypothyroidism usually does not occur in patients who are euthyroid postoperatively. Therefore, annual evaluation of serum TOSH is satisfactory as follow-up. However, the fact that patients can still develop hypothyroidism later than 12 months after surgery cannot be ignored, even though such cases were not found in our study. Thus, further follow-up of thyroid function is required, particularly for patients who have risk factors for hypothyroidism.

A high preoperative TOSH level is the most frequently observed risk factor in patients who develop postoperative hypothyroidism (11–14), likely reflecting an already compromised level of thyroid function. Therefore, a high preoperative TOSH level, which is universally accepted as a risk factor in adults, is also an indicator of risk in children. Our results demonstrated that patients with hypothyroidism after surgery had significantly higher levels of TOSH than did patients with normal thyroid function. As preoperative TOSH levels (>2.2 μIU/ml) appear to indicate a low thyroid functional reserve in these eight patients, the thyroid function of these patients after surgery is insufficient for maintenance, and hypothyroidism may readily develop.

In this study, most of the patients are teenage girls, the increase of anti-Tg in adolescent girls seems to be a common phenomenon. Estrogen's induction of the immune system in early adolescence was thought to be the main reason for this effect (15). Anti-Tg is a parameter related to age and puberty, and the concentration is higher in girls (16). The degree of thyroid lymphocytic infiltration during surgery has also been noted as a possible predictor of hypothyroidism. The hypothesis that lymphocyte infiltration is an important risk factor for hypothyroidism is also supported by our results (2, 14, 17). The presence of lymphocytes in thyroid tissue may indicate active or persistent disease, so thyroid function may decline gradually. Berglund et al. (9) reported that 33% of patients with significant thyroid lymphocytic infiltration develop postoperative biochemical hypothyroidism, but only 4% of patients with mild infiltration develop hypothyroidism. Nonetheless, that study did not definitively quantify the increased risk associated with lymphocytic infiltration. In our study, the degree of lymphocytic infiltration was associated with the development of postoperative hypothyroidism, and lymphocytic infiltration of grade 3 or 4 was significantly associated. In contrast, in the absence of significant lymphocytic infiltration, the risk of developing subsequent hypothyroidism is rather low. In the present study, there was only one patient who developed hypothyroidism without lymphocytic infiltration; remarkably, this patient had relatively high levels of TOSH before surgery.

Thyroid dysfunction following hemithyroidectomy can negatively affect growth and brain maturation in infancy and childhood, and the clinical outcome varies with age and the severity of thyroid impairment. The effects of thyroid hormones on growth, skeletal maturation and cognitive development in adolescent should be considered (18). The beneficial effects of L-T4 on the clinical symptoms and signs of hypothyroidism and goiter, especially in adolescent with Hashimoto's thyroiditis, have been confirmed in many studies (19–21). Regardless, long-term research on the impact of L-T4 on other clinical outcomes, particularly in children with non-autoimmune subclinical hypothyroidism, is still lacking, and the few non-randomized short-term trials to date have reported inconsistent results. Moreover, the adverse effects of thyroid hormone replacement therapy have recently received growing attention as a major issue in the management of subclinical thyroid disorders, and the negative effects of treatment on the cardiovascular and skeletal systems were found to be greater than expected (22–25). De Luca et al. (26) discussed the indications for L-T4 treatment in the literature review of asymptomatic hypothyroidism in children. These authors do not recommend hormone replacement unless TOSH levels remain between 5 and 10 IU/ml, except in the presence of underlying disease, such as goiter or nodules, or anti-thyroid antibody positivity, all of which are factors of risk for thyroid dysfunction. The L-T4 treatment and no treatment in children with mild and idiopathic subclinical hypothyroidism had no correlation with the subsequent evolution of growth parameters (27). Traditionally, the hypothyroidism that develops after hemithyroidectomy has been incorrectly assumed to be permanent. Hence, most patients who develop hypothyroidism after surgery receive thyroid hormone replacement therapy without any consideration for natural recovery, even in cases of subclinical hypothyroidism with mild TOSH elevation (2, 14, 28). In addition, once thyroid hormone replacement is initiated, it is unlikely that it will be discontinued for an evaluation of whether the remaining thyroid function is adequate to maintain the euthyroid state (14). In our study, patients with asymptomatic mild postoperative hypothyroidism were followed up without immediate thyroid hormone replacement. Four of these patients with subclinical hypothyroidism spontaneously recovered to euthyroidism within 12 months. These findings indicate that serum TOSH elevation might be a transient compensatory phenomenon that occurs during the early postoperative period that can be followed up closely without immediate treatment for 12 months after the development of hypothyroidism. Furthermore, these results suggest that the pituitary-thyroid axis undergoes an adaptation process regardless of the cause of hypothyroidism, including when hypothyroidism is due to abrupt surgical loss of thyroid tissue, especially in children and adolescents. Therefore, thyroid hormone replacement for subclinical hypothyroidism that develops after hemithyroidectomy in pediatric patients should be applied in a more prudent manner (29).

In view of the retrospective characteristics of this study, it is hard to entirely describe the progression of thyroid function or dysfunction following hemithyroidectomy in young patients. However, our study does suggest that some patients may develop symptomatic or biochemical hypothyroidism that will resolve spontaneously in some cases. Preoperative serum TOSH levels and lymphocytic infiltration are helpful in predicting hypothyroidism, and this information may help in the design of a method for predicting hypothyroidism and factors that affect its development following hemithyroidectomy. Pathologists should systematically examine the degree of lymphocytic infiltration following hemithyroidectomy.

## Conclusions

In conclusion, our results show that approximately one-quarter of pediatric patients undergoing hemithyroidectomy will suffer from hypothyroidism as a direct outcome of surgery. In this study, however, thyroid hormone replacement was required in only 16.28% of hemithyroidectomy cases. To ensure that pediatric cases of postoperative hypothyroidism are detected, we recommend periodically performing regular thyroid function measurements for life after surgery in children and adolescents who undergo hemithyroidectomy. Moreover, evaluation of hypothyroidism after surgery should be performed quarterly during the first 12 months and then, for example, annually. The presence of an elevated preoperative TOSH level and lymphocytic infiltration, particularly a preoperative TOSH level of at least 2.2 μIU/ml and lymphocytic infiltration graded 3 or 4, was found to be strongly predictive of hypothyroidism after hemithyroidectomy. Mild postoperative subclinical hypothyroidism following hemithyroidectomy in children and adolescents can be followed up without immediate L-T4 replacement with the expectation of recovery.

## Data Availability Statement

The datasets analyzed in this manuscript are not publicly available. Request to access the datasets should be directed to yangjun@xinhuamed.com.cn.

## Ethics Statement

The studies involving human participants were reviewed and approved by Shanghai Jiao Tong University Research Compliance Office. Written informed consent to participate in this study was provided by the participants' legal guardian/next of kin. Informed consent was obtained from all individual participants included in the study. Institutional review board approval was obtained from the Shanghai Jiao Tong University Research Compliance Office. Informed consent was obtained from the parents of all children.

## Author Contributions

JC analyzed the data and drafted the manuscript. SH who contributed equally to JC in this work. XL contributed to design the article. JY contributed to conception and designing the article, gave final approval and agreed to be accountable for all aspects of work ensuring integrity and accuracy.

### Conflict of Interest

The authors declare that the research was conducted in the absence of any commercial or financial relationships that could be construed as a potential conflict of interest.

## Abbreviations

TSH: Thyrotropin
L-T4: Levothyroxine
FT4: Free Thyroxine
T4: Thyroxine
T3: Triiodothyronine
anti-Tg: Anti-thyroglobulin antibody
anti-TPO: Anti-peroxidase antibody.
